# Incidence trends and disparities in *Helicobacter pylori* related malignancy among US adults, 2000–2019

**DOI:** 10.3389/fpubh.2022.1056157

**Published:** 2022-11-28

**Authors:** Yafang Lai, Haoting Shi, Zixin Wang, Yibo Feng, Yujia Bao, Yongxuan Li, Jinhui Li, Anhao Wu

**Affiliations:** ^1^Kunming Maternal and Child Health Service Centre, Kunming City Maternal and Child Health Hospital, Kunming, China; ^2^Department of Radiation Therapy, Ruijin Hospital, Shanghai Jiao Tong University School of Medicine, Shanghai, China; ^3^School of Public Health, Weifang Medical University, Weifang, China; ^4^School of Medicine, Hangzhou Normal University, Hangzhou, China; ^5^Weifang Medical University, Weifang, China; ^6^Department of Urology, Stanford University Medical Center, Stanford, CA, United States; ^7^Department of Mammary Surgery I, The Third Affiliated Hospital of Kunming Medical University, Kunming, China

**Keywords:** gastric cancer, gastric non-Hodgkin lymphoma, incidence, *H. pylori*, trend

## Abstract

**Background:**

*Helicobacter pylori* (*H. pylori*) is closely related to the carcinogenesis of gastric cancer (GC) and gastric non-Hodgkin lymphoma (NHL). However, the systemic trend analysis in *H. pylori*-related malignancy is limited. We aimed to determine the national incidence trend in non-cardia GC, cardia GC, and gastric NHL in the US during 2000–2019.

**Method:**

In this population-based study, we included 186,769 patients with a newly diagnosed *H. pylori*-related malignancy, including non-cardia GC, cardia GC, and gastric NHL from the Surveillance, Epidemiology, and End Results (SEER) Registry from January 1, 2000 to December 31, 2019. We determined the age-adjusted incidence of three *H. pylori*-related malignancies respectively. Average annual percentage change (AAPC) in 2000–2019 was calculated to describe the incidence trends. Analyses were stratified by sex, age, race and ethnicity, geographic location and SEER registries. We also determined the 5-year incidence (during 2015–2019) by SEER registries to examine the geographic variance.

**Results:**

The incidence in non-cardia GC and gastric NHL significantly decreased during 2000–2019, while the rate plateaued for cardia GC (AAPCs, −1.0% [95% CI, −1.1%−0.9%], −2.6% [95% CI, −2.9%−2.3%], and −0.2% [95% CI, −0.7%−0.3%], respectively). For non-cardia GC, the incidence significantly increased among individuals aged 20–64 years (AAPC, 0.8% [95% CI, 0.6–1.0%]). A relative slower decline in incidence was also observed for women (AAPC, −0.4% [95% CI, −0.6%−0.2%], *P* for interaction < 0.05). The incidence of cardia GC reduced dramatically among Hispanics (AAPC, −0.8% [95% CI, −1.4%−0.3%]), however it increased significantly among nonmetropolitan residents (AAPC, 0.8% [95% CI, 0.4–1.3%]). For gastric NHL, the decreasing incidence were significantly slower for those aged 20–64 years (AAPC, −1.5% [95% CI, −1.9–1.1%]) and Black individuals (AAPC, −1.3% [95% CI, −1.9–1.1%]). Additionally, the highest incidence was observed among Asian and the Black for non-cardia GC, while Whites had the highest incidence of cardia GC and Hispanics had the highest incidence of gastric NHL (incidence rate, 8.0, 8.0, 3.1, and 1.2, respectively) in 2019. Geographic variance in incidence rates and trends were observed for all three *H. pylori*-related malignancies. The geographic disparities were more pronounced for non-cardia GC, with the most rapid decline occurring in Hawaii (AAPC, −4.5% [95% CI, −5.5–3.6%]) and a constant trend in New York (AAPC 0.0% [95% CI, −0.4–0.4%]), the highest incidence in Alaska Natives, and the lowest incidence among Iowans (14.3 and 2.3, respectively).

**Conclusion:**

The incidence of *H. pylori*-related cancer declined dramatically in the US between 2000 and 2019, with the exception of cardia GC. For young people, a rising trend in non-cardia GC was noted. Existence of racial/ethnic difference and geographic diversity persists. More cost-effective strategies of detection and management for *H. pylori* are still in demand.

## Introduction

*Helicobacter pylori* (*H. pylori*) is the most well-described risk factor for gastric cancer (GC), and recognized as one of the pathogens for gastric non-Hodgkin lymphoma (NHL) (1, 2). In 2014, *H. pylori* approximately infects 50% of the global population (3). The carcinogenic process is mainly due to its virulence factors including urease, flagella, VacA, and CagA (4, 5) *H. pylori* infection causes chronic inflammation, which leads to precancerous alterations such as gastritis and intestinal metaplasia. These changes eventually develop to gastric epithelial neoplasms, termed GC (5). GC can be classified into two subtypes, cardia and non-cardia, based on their anatomic sites. *H. pylori* is more relevant to non-cardia GC, accounting for 90% of cases of the non-cardia GC, while a portion of cardia GC can also be associated with *H. pylori* atrophic gastritis (6). It has been clearly established that the eradication of *H. pylori* could prevent and even reverse the carcinogenic process of GC (7, 8).

GC is currently the fifth most frequent cancer and the fourth leading cause of cancer-related death (9). Despite the decrease in incidence and mortality rates in past decades (10), it remains to be a vital contributor to the global burden of cancer. Additionally, NHLs, also known as malignant proliferations of lymphoid cells, are the most common hematological malignancies worldwide (9). NHLs have been on the rise globally during the last few decades (11). The colonization of *H. pylori* in gastric mucosa may result in the interaction of bacteria and B-cell, followed by the formation of lymphoid follicles and, finally NHL in the gastric location (1, 12). The age- and gender-adjusted annual incidence of gastrointestinal NHL was 1.73 per 100,000 among North American population in 2008 (13).

A study using attributable fractions (AF) to describe the correlation between specific anatomical cancer sites and relevant infectious pathogens showed that AF of *H. pylori* was 89% to non-cardia GC, 29% to cardia GC and 74% to gastric NHL (14). However, systemic trend analysis of *H. pylori*-related malignancy remains inadequately characterized. Lack of timely monitoring might impede the formulation of public health policies for *H. pylori* infection prevention and management, as well as the allocation of the clinical and public health resources for *H. pylori*-related malignancy management. It is crucial to update the surveillance of the trends in malignancies caused by *H. pylori*. To decrease the global burden of *H. pylori*-related cancers, we aimed to determine the trend of cardia and non-cardia gastric cancer and gastric NHLs incidence at the national level in order to provide a reference for better prevention and resource allocation.

## Materials and methods

### Data source and case identification

In this study, GC (including non-cardia and cardia) and gastric NHL cases recorded during 2000–2019 in 22 SEER registries (https://seer.cancer.gov/) were included, which represents ~47.9% of the US population. The SEER 22 registry includes San Francisco-Oakland, Connecticut, Hawaii, Iowa, New Mexico, Seattle (Puget Sound), Utah, Atlanta (Metropolitan), San Jose-Monterey, Los Angeles, Alaska Natives, Rural Georgia, Greater California, Kentucky, Louisiana, New Jersey, Greater Georgia, Idaho, New York, Massachusetts, Illinois, and Texas tumor registries.

Cases were identified by the International Classification of Diseases for Oncology (ICD-O-3, third edition). GC was identified by topographic code C16.0 to C16.6 (excluding histology code 9050–9055, 9140, 9590–9992), where cardia GC were confirmed using ICD-O-3 topographic code C16.0 and non-cardia GC were confirmed using ICD-O-3 topographic code C16.1 to C16.6. Gastric NHL was identified by topographic code C16.0 to C16.9 and the corresponding histology code (detailed in the Supplementary eMethod). Only the first diagnosed cancer of each individual was included. Cases with age at diagnosis <20 years were excluded. The study was exempted from ethical review because the data were publicly available and de-identified. This study follows the Strengthening the Reporting of Observational Studies in Epidemiology (STROBE) reporting guideline.

### Measurements and variables

Data on annual incidence rates in non-cardia GC, cardia GC, and gastric NHL were obtained from 22 SEER registries database respectively. The incidence rate (age-adjusted to 2000 U.S. standard population) per 100,000 population with a 95% confidence interval (CI) were estimated and adjusted to reporting delay [by race and cancer type (15)]. The trends in incidence rate were quantified by average annual percentage changes (AAPCs) in 2000–2009, 2010–2019 and 2000–2019, where AAPCs during 2000–2019 were used to justify the trend.

Further stratified analyses for incidence trends were performed by age [grouped as young people (20–64 years) and the elderly (65 years or older)], race/ethnicity (grouped as non-Hispanic White, non-Hispanic Black, Hispanic and non-Hispanic Asia/Pacific Islander, here after, White, Black, Hispanic and Asian, respectively), geographic location (categorized as metropolitan and nonmetropolitan), and 22 SEER registries. Of note, non-Hispanic American Indian and Alaska Natives were not included when stratified by race/ethnicity due to the small sample size. We also estimated the contemporaneous five-year incidence with 95% CIs (15) of three cancers by SEER registries to compare the geographic variance in incidence.

### Statistical analysis

The incidence was calculated using SEER^*^Stat version 8.4.0 (National Cancer Institute). According to the recommendation, when the case number was <16, the incidence was not estimated due to the small sample size. AAPCs with 95% CIs were generated using Joinpoint Regression Analysis software version 4.9.1.0 (National Cancer Institute). We examined the differences in these two AAPCs by Student's *t*-test to detect the change in incidence trends over time. The statistical significance of AAPC estimates or their interactive changes was examined by *t-*test, and a two-sided *P* < 0.05 was considered statistically significant. Of note, multiple testing correction was used for groups with AAPCs of significance. Two sensitivity analyses were conducted. We first used SEER 17 database to estimate the incidence trend. Then, we included GC with site code C16.8 (Overlapping lesion of stomach) and C16.9 (Stomach, NOS) into non-cardia GC to determine the trend. A sensitivity analysis was conducted using SEER 17 database to estimate the incidence trend. Data were analyzed from 1 January 2000 to 31 December 2019.

## Results

From 2000 through 2019, a total of 186,769 *H. pylori*-related cancer cases were identified in the SEER database, of which 109,469 (58.6%) were White, 23,409 (12.5%) were Black, 32,752 (17.5%) were Hispanic, and 19,541 (10.5%) were Asian. Among those cases, 115,666 (61.9%) were male and 115,514 (61.8%) were of age 65 years or older. Overall, the incidence significantly decreased during 2000–2019 for non-cardia GC (AAPC, −1.0% [95% CI, −1.1 to −0.9%]) and gastric NHL (AAPC, −2.6% [95% CI, −2.9 to −2.3%]), whereas cardia GC levels has plateaued (AAPC, −0.2% [95% CI, −0.7 to −0.3%]). Sensitivity analyses showed the robustness of our findings (Supplementary eResult and eTable 1).

### Trend in non-cardia GC incidence

**Figure 1:**
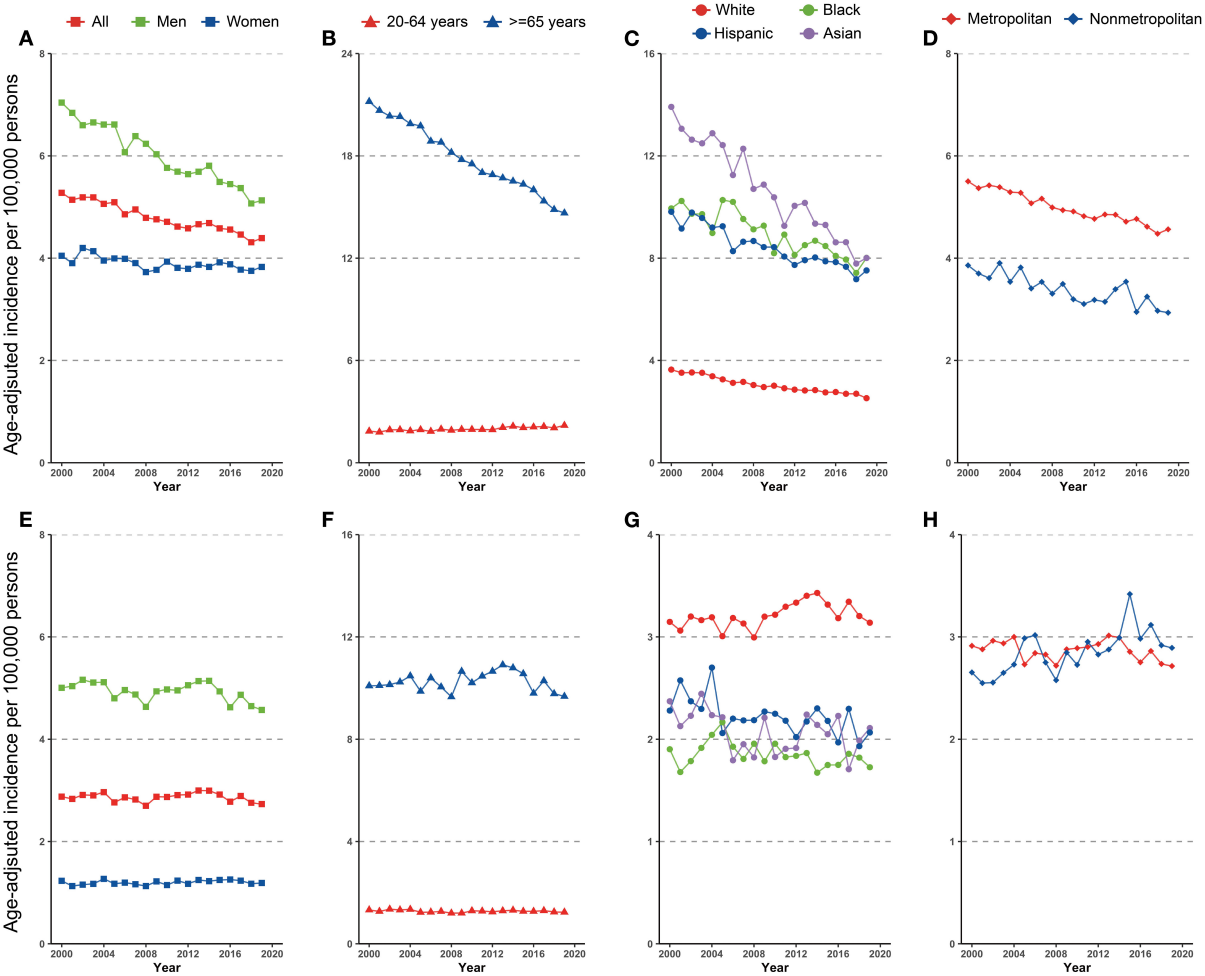
Incidence trend in gastric cancer among US adults, 2000–2019 **(A–D)** cardia gastric cancer by sex, age, race/ethnicity, and geographic location **(E–H)** non-cardia gastric cancer by sex, age, race/ethnicity, and geographic location.

A total of 101,198 non-cardia GC were reported during 2000–2019, of whom 55,256 (54.6%) were men, 45,697 (45.2%) were White 17,326 (17.1%) were Black, 22,409 (22.1%) were Hispanic, and 14,819 (14.6%) were Asian. Trends in non-cardia GC incidence in the overall population and subpopulations are presented in Figures 1A–D and Table 1.

**Table 1:** Incidence rate and trend in non-cardia gastric cancer among US adults aged ≥20, 2000–2019.

	**Case number No. %**	**Incidence rate, 2000 (95% CI)**	**Incidence rate, 2019 (95% CI)**	**Average APC, 2000-2009 (95% CI)**	**Average APC, 2010-2019 (95% CI)**	**Average APC, 2000–2019 (95% CI)**
**Overall**	101,198	5.3 (5.1–5.4)	4.4 (4.3–4.5)	−1.0^*^ (−1.1 to −0.9)	−1.0^*^ (−1.1 to −0.9)	−1.0^*^ (−1.1 to −0.9)
**Sex**
Men	55,256 (54.6)	7.0 (6.8–7.3)	5.1 (4.9–5.3)	−1.6^*^ (−1.8 to −1.4)	−1.6^*^ (−1.8 to −1.4)	−1.6^*^ (−1.8 to −1.4)
Women	45,942 (45.4)	4.0 (3.9–4.2)	3.8 (3.7–4.0)	−0.4^*^ (−0.6 to −0.2)	−0.4^*^ (−0.6 to −0.2)	−0.4^*^ (−0.6 to −0.2)
**Age**
20-64	37,301 (36.9)	1.9 (1.8–2.0)	2.2 (2.1–2.3)	0.8^*^ (0.6 to 1.0)	0.8^*^ (0.6 to 1.0)	0.8^*^ (0.6 to 1.0)
≥65	63,897 (63.1)	21.2 (20.5–21.9)	14.6 (14.1–15.2)	−1.8^*^ (−2.2 to −1.5)	−2.0^*^ (−2.5 to −1.4)	−1.9^*^ (−2.3 to −1.6)
**Race/ethnicity^#^**
Non-Hispanic white^§^	45,697 (45.2)	3.6 (3.5–3.8)	2.5 (2.4–2.6)	−2.2^*^ (−2.6 to −1.7)	−1.4^*^ (−1.8 to −1.1)	−1.8^*^ (−2.1 to −1.5)
Non-Hispanic black	17,326 (17.1)	9.9 (9.2–10.7)	8.0 (7.5–8.5)	−1.5^*^ (−1.8 to −1.1)	−1.5^*^ (−1.8 to −1.1)	−1.5^*^ (−1.8 to −1.1)
Hispanic	22,409 (22.1)	9.8 (9.1–10.6)	7.5 (7.1–7.9)	−1.4^*^ (−1.7 to −1.2)	−1.4^*^ (−1.7 to −1.2)	−1.4^*^ (−1.7 to −1.2)
Non-Hispanic API	14,819 (14.6)	13.9 (12.8–15.1)	8.0 (7.5–8.6)	−2.8^*^ (−3.2 to −2.5)	−2.8^*^ (−3.2 to −2.5)	−2.8^*^ (−3.2 to −2.5)
**Geographic location**
Metropolitan	92,142 (91.1)	5.5 (5.3–5.7)	4.6 (4.4–4.7)	−1.0^*^ (−1.1 to −0.9)	−1.0^*^ (−1.1 to −0.9)	−1.0^*^ (−1.1 to −0.9)
Nonmetropolitan	8,852 (8.7)	3.9 (3.5–4.2)	2.9 (2.6–3.2)	−1.2^*^ (−1.6 to −0.8)	−1.2^*^ (−1.6 to −0.8)	−1.2^*^ (−1.6 to −0.8)

APC, annual percentage change; API, Asian and Pacific Islander.

* Indicates the average APC was significantly different from zero.

# The stratified analysis by race/ethnicity was limited among Non-Hispanic White, Non-Hispanic Black, Hispanic and Non-Hispanic API.

§ Indicates that the average APCs of 2000–2009 and 2010–2019 was significantly different.

Disparities were observed for non-cardia GC incidence when stratified by sex and age. The incidence of non-cardia GC decreased dramatically in both men and women, although the fall in men was significantly more rapid than in women (AAPCs, −1.6% [95% CI, −1.8 to −1.4%] and −0.4% [95% CI, −0.6 to −0.2%], *P* for interaction < 0.05, Figure 1A and Table 1). The significant decrease was limited in individuals aged ≥65 years (AAPC, −1.9% [95% CI, −2.3 to 1.6%]) and significantly increased for individuals aged 20–64 years (AAPC, 0.8% [95% CI, 0.6 to 1.0%]) (Figure 1B and Table 1).

Non-cardia GC incidence significantly decreased among all racial and ethnicities subgroups (Figure 1C), with an AAPC ranging from −2.8% (95% CI, −3.2 to −2.5%) for Asians to −1.4% (95% CI, −1.7 to −1.2%) for Hispanics. Notably, Asians had the greatest incidence in 2019 (8.0 [95% CI, 7.5–8.6]), whereas Blacks had the same incidence in 2019 (8.0 [95% CI, 7.5–8.5]).

Similar incidence decreases were observed in both metropolitan and nonmetropolitan areas, with a greater incidence in metropolitan areas (Figure 1D and Table 1). Nonetheless, SEER registries' stratification study revealed geographical variation and gender discrepancies. The fastest decline was observed in Hawaiis for both sexes (AAPCs, −4.9% [95% CI, −6.1 to −3.7%] and −3.9% [95% CI, −5.4 to −2.5%] for men and women, respectively), while the trend plateaued in Atlanta (Metropolitan), San Jose-Monterey, Kentucky, Idaho, New York, Illinois, and Texas (eTable 2). Notably, among those registries with stable trends (with the exception of Idaho, where AAPC did not estimate by sex due to the small sample size), a significant drop was detected for men but not for women. Also, a significant fall in non-cardia incidence was not observed for women in majority of the registries, despite a considerable decline in the overall trend (eTable 2). In addition, the highest incidence rate in 2015–2019 was identified in Alaska Natives for both sexes (**Figure 3A** and eTable 2).

### Trend in cardia GC incidence

During 2000–2019, a total of 61,789 cardia GC were reported, of which 47,516 (76.9%) were male and 48,496 (78.5%) were White, 3,753 (6.1%) were Black, 6,113 (9.9%) were Hispanic, and 3,044 (4.9%) were Asian. Trends in cardia GC incidence in the whole population and subpopulations are shown in Figures 1E–H and Table 2. Similar to the incidence pattern in the general population, the trend in cardia GC remained consistent in the majority of the subpopulations, but significantly decreased among Hispanics (AAPC, −0.8% [95% CI, −1.4 to −0.3%]). Contrary to non-cardia GC, the highest incidence of cardia GC was observed among Whites rather than Asians in 2019 among all races and ethnicities.

**Table 2:** Incidence rate and trend in cardia gastric cancer among US adults aged ≥20, 2000–2019.

	**Case Number No. %**	**Incidence rate, 2000 (95% CI)**	**Incidence rate, 2019 (95% CI)**	**Average APC, 2000-2009 (95% CI)**	**Average APC, 2010-2019 (95% CI)**	**Average APC, 2000–2019 (95% CI)**
**Overall**	61,789	2.9 (2.8–3.0)	2.7 (2.6–2.8)	0.2 (−0.1 to 0.6)	−0.6 (−1.5 to 0.2)	−0.2 (−0.7 to 0.3)
**Sex**						
Men	47,516 (76.9)	5.0 (4.8–5.2)	4.6 (4.4–4.8)	−0.6 (−1.3 to 0.1)	−0.6 (−1.8 to 0.1)	−0.6 (−1.3 to 0.1)
Women	14,273 (23.1)	1.2 (1.1–1.3)	1.2 (1.1–1.3)	0.2 (−0.1 to 0.5)	0.2 (−0.1 to 0.5)	0.2 (−0.1 to 0.5)
**Age**						
20–64	24,683 (39.9)	1.3 (1.2–1.4)	1.2 (1.2–1.3)	−0.2 (−0.4 to 0.1)	−0.2 (−0.4 to 0.1)	−0.2 (−0.4 to 0.1)
≥65^§^	37,106 (60.1)	10.1 (9.6–10.6)	9.7 (9.3–10.1)	0.5^*^ (0.0 to 0.9)	−0.9 (−1.8 to 0.0)	−0.2 (−0.7 to 0.3)
**Race/ethnicity^#^**						
Non-Hispanic White	48,496 (78.5)	3.1 (3.0–3.3)	3.1 (3.0–3.3)	0.0 (−0.6 to 0.6)	−0.2 (−1.1 to 0.7)	0.0 (−0.6 to 0.7)
Non-Hispanic Black	3,753 (6.1)	1.9 (1.6–2.2)	1.7 (1.5–2.0)	−0.5 (−0.9 to 0.0)	−0.5 (−0.9 to 0.0)	−0.5 (−0.9 to 0.0)
Hispanic	6,113 (9.9)	2.3 (1.9–2.7)	2.1 (1.9–2.3)	−0.8^*^ (−1.4 to −0.3)	−0.8^*^ (−1.4 to −0.3)	−0.8^*^ (−1.4 to −0.3)
Non-Hispanic API	3,044 (4.9)	2.4 (1.9–2.9)	2.1 (1.8–2.4)	−0.5 (−1.3 to 0.3)	−0.5 (−1.3 to 0.3)	−0.5 (−1.3 to 0.3)
**Geographic location**						
Metropolitan	54,098 (87.6)	2.9 (2.8–3.0)	2.7 (2.6–2.8)	−0.5 (−1.2 to 0.2)	−0.7 (−1.6 to 0.3)	−0.5 (−1.2 to 0.2)
Nonmetropolitan	7,624 (12.3)	2.7 (2.4–3.0)	2.9 (2.6–3.2)	0.8^*^ (0.4 to 1.3)	0.8^*^ (0.4 to 1.3)	0.8^*^ (0.4 to 1.3)

APC, annual percentage change; API, Asian and Pacific Islander.

* Indicates the average APC was significantly different from zero.

# The stratified analysis by race/ethnicity was limited among Non-Hispanic White, Non-Hispanic Black, Hispanic and Non-Hispanic API.

§ Indicates that the average APCs of 2000–2009 and 2010–2019 was significantly different.

However, a significant increase was noted among those who lived in nonmetropolitan areas (AAPC, 0.8% [95% CI, 0.4 to 1.3%]). Though in most registries, the cardia GC incidence remained stable or decreased, a significant increase was observed in Kentucky and Greater Georgia (AAPCs, 2.4% [95% CI 1.3 to 3.4%] and 1.0% [95% CI 0.2 to 1.8%], respectively). Additionally, the incidence significantly increased for women but not for men in Massachusetts and Illinois (AAPCs, 2.1% [95% CI, 0.8 to 3.4%] and 1.7% [95% CI 0.7 to 2.7%], respectively). The highest incidence rate in 2015–2019 was found in Connecticut for both sexes (**Figure 3B** and eTable 3).

### Trend in gastric NHL incidence

**Figure 2:**
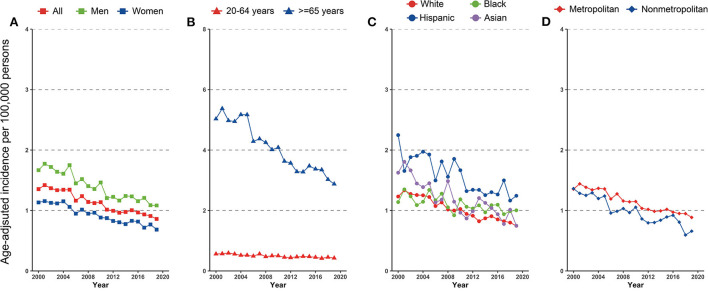
Incidence trend in gastric non-Hodgkin lymphoma by **(A)** sex, **(B)** age, **(C)** race/ethnicity, and **(D)** geographic location among US adults, 2000–2019.

A total of 23,782 gastric NHL were identified during 2000–2019, of which 12,894 (54.2%) were men, 15,276 (64.2%) were White, 2,330 (9.8%) were Black, 4,230 (17.8%) were Hispanic, and 1,678 (7.1%) were Asian. Trends in cardia gastric NHL incidence in the overall population and subpopulations were shown in Figure 2 and Table 3. When stratified by age, the discrepancy was seen, but not by gender. Although a substantial fall was detected for both 20–64-year-olds and those 65 or older, the loss for the latter group was significantly more rapid (AAPCs, −1.5% [95% CI, −1.9 to −1.1%] and −3.2% [95% CI, −3.6 to −2.8%], respectively, *P* for interaction < 0.001). The incidence of gastric NHL reduced equally in men and women (−2.6% [95% CI, −3.1 to −2.2%] and −2.7% [95% CI, −3.0 to −2.3%], *P* for interaction > 0.05).

**Table 3:** Incidence rate and trend in gastric non-Hodgkin lymphoma among US adults aged ≥20, 2000–2019.

	**Case number No. %**	**Incidence rate, 2000 (95% CI)**	**Incidence rate, 2019 (95% CI)**	**Average APC, 2000-2009 (95% CI)**	**Average APC, 2010-2019 (95% CI)**	**Average APC, 2000–2019 (95% CI)**
**Overall**	23,782	1.4 (1.3–1.4)	0.9 (0.8–0.9)	−2.6^*^ (−2.9 to −2.3)	−2.6^*^ (−2.9 to −2.3)	−2.6^*^ (−2.9 to −2.3)
**Sex**						
Men	12,894 (54.2)	1.7 (1.5–1.8)	1.1 (1.0–1.2)	−2.6^*^ (−3.1 to −2.2)	−2.6^*^ (−3.1 to −2.2)	−2.6^*^ (−3.1 to −2.2)
Women	10,888 (45.8)	1.1 (1.0–1.2)	0.7 (0.6–0.8)	−2.7^*^ (−3.0 to −2.3)	−2.7^*^ (−3.0 to −2.3)	−2.7^*^ (−3.0 to −2.3)
**Age**						
20–64	9,271 (39.0)	0.6 (0.5–0.6)	0.4 (0.4–0.5)	−1.5^*^ (−1.9 to −1.1)	−1.5^*^ (−1.9 to −1.1)	−1.5^*^ (−1.9 to −1.1)
≥65	14,511 (61.0)	5.0 (4.7–5.4)	2.9 (2.7–3.1)	−3.2^*^ (−3.6 to −2.8)	−3.2^*^ (−3.6 to −2.8)	−3.2^*^ (−3.6 to −2.8)
**Race/ethnicity^#^**						
Non-Hispanic white	15,276 (64.2)	1.2 (1.1–1.3)	0.7 (0.7–0.8)	−2.9^*^ (−3.3 to −2.6)	−2.9^*^ (−3.3 to −2.6)	−2.9^*^ (−3.3 to −2.6)
Non-Hispanic black	2,330 (9.8)	1.1 (0.9–1.4)	1.0 (0.8–1.2)	−1.3^*^ (−1.9 to −0.6)	−1.3^*^ (−1.9 to −0.6)	−1.3^*^ (−1.9 to −0.6)
Hispanic	4,230 (17.8)	2.2 (1.9–2.6)	1.2 (1.1–1.4)	−2.8^*^ (−3.6 to −2.0)	−2.8^*^ (−3.6 to −2.0)	−2.8^*^ (−3.6 to −2.0)
Non-Hispanic API	1,678 (7.1)	1.6 (1.3–2.0)	0.7 (0.6–0.9)	−3.6^*^ (−4.6 to −2.6)	−3.6^*^ (−4.6 to −2.6)	−3.6^*^ (−4.6 to −2.6)
**Geographic location**						
Metropolitan	21,189 (89.1)	1.4 (1.3–1.4)	0.9 (0.8–0.9)	−2.5^*^ (−2.8 to −2.2)	−2.5^*^ (−2.8 to −2.2)	−2.5^*^ (−2.8 to −2.2)
Nonmetropolitan	2,579 (10.8)	1.4 (1.2–1.6)	0.7 (0.5–0.8)	−3.4^*^ (−4.1 to −2.7)	−3.4^*^ (−4.1 to −2.7)	−3.4^*^ (−4.1 to −2.7)

APC, annual percentage change; API, Asian and Pacific Islander.

* Indicates the average APC was significantly different from zero.

# The stratified analysis by race/ethnicity was limited among Non-Hispanic White, Non-Hispanic Black, Hispanic and Non-Hispanic API

When segregated by race and ethnicity, a mild discrepancy was identified. Although the prevalence declined dramatically in all racial and ethnic subpopulations, Asians experienced a comparatively faster decline (AAPC, −3.6% [95% CI, −4.6 to −2.6%]) and a slowest rate for Blacks (AAPC, −1.3% [95% CI, −1.9 to −1.1%], compared with White, *P* for interaction < 0.001).

**Figure 3:**
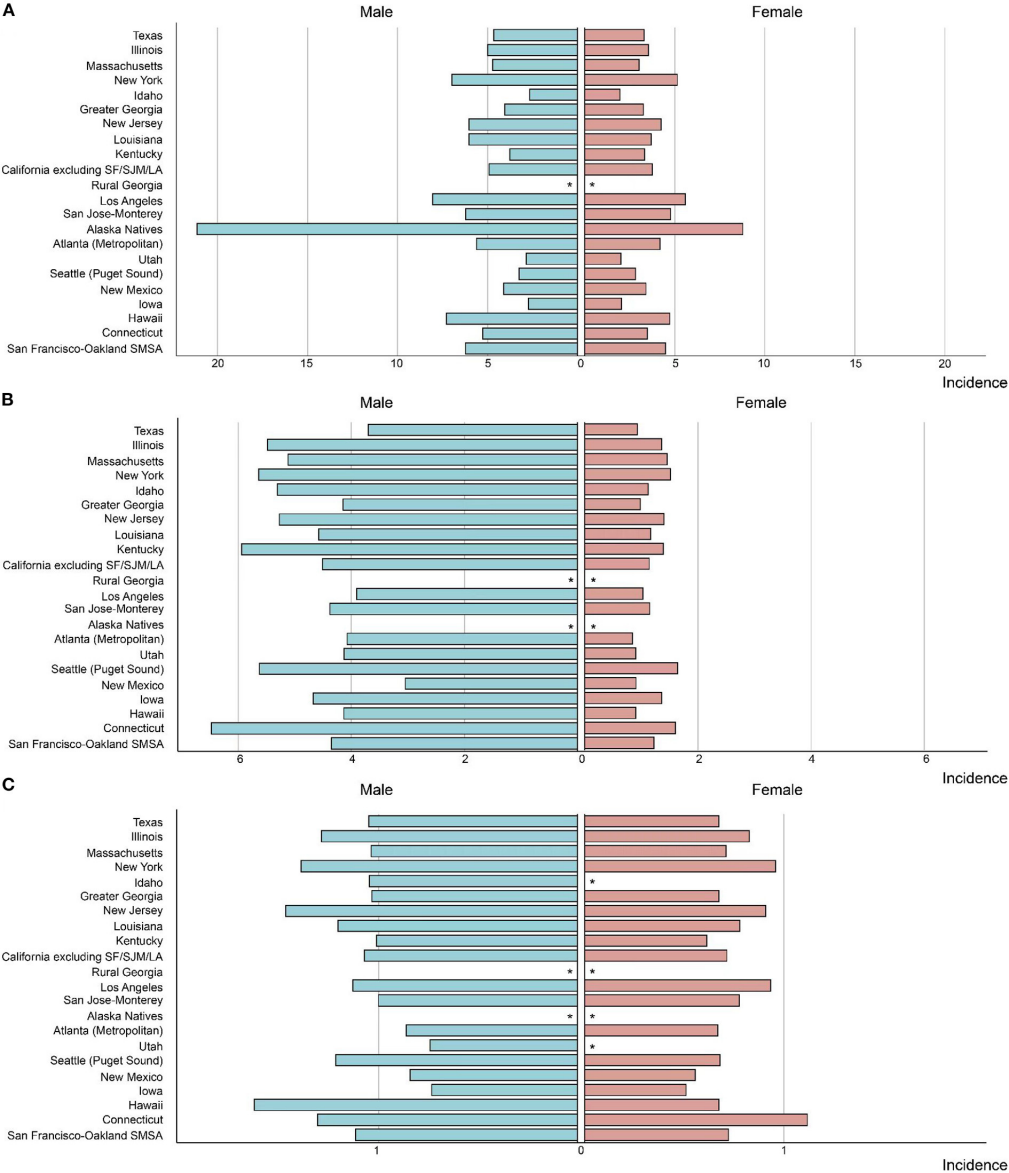
The incidence rate in *Helicobacter pylori* related malignancy among US adults by SEER registry and sex, 2015–2019 **(A)** non-cardia gastric cancer, **(B)** cardia gastric cancer, **(C)** gastric non-Hodgkin lymphoma. “^*^” indicates that the number of observed cases was <16 and the incidence was not estimated due to small sample size.

Differences also occurred for the gastric NHL incidence when stratified by geographic location. Among those lived in metropolitan, the gastric NHL incidence decreased significantly slower than those in nonmetropolitan area (AAPCs, −2.5% [95% CI, −2.8 to −2.2%] and −3.4% [95%CI, −4.1 to −2.7%], respectively, *P* for interaction = 0.021, Figure 2D and Table 3). Highest incidence rate for overall population and females were found in Connecticut in 2015–2019, along with a stable overall trend (AAPC, −1.1% [95% CI, −2.9% to 0.7%]) (Figure 3C and eTable 3). Furthermore, Hawaii has the highest gastric NHL incidence rate for men between 2015 and 2019 (Figure 3C and eTable 4).

## Discussion

In this study, we found that during 2000–2019, the overall incidence trend of *H. pylori*-related malignancy among adults in the United States fell dramatically significantly, except for cardia GC that remained relatively stable. For non-cardia GC, the incidence declined more slowly for women and increased among younger adults. The decrease in incidence of cardia GC in Hispanic individuals is limited. For gastric NHL, the decrement in incidence was slower in younger adults and Blacks. Moreover, Asians had the highest incidence of non-cardia GC, while Whites had the highest incidence of cardia GC and Hispanics had the highest incidence of gastric NHL. Geographic variation in incidence rates and trends was observed for all three *H. pylori*-related cancers, with non-cardia GC being the most prominent.

The decreasing trend in incidence for non-cardia GC and gastric NHL may be related to the control of *H. pylori*, as *H. pylori* is a well-known determining factor for non-cardia GC (16, 17). First, the secondary prevention of *H. pylori* spreading contributed to the decreasing incidence, especially the improvements in sanitary conditions, which significantly limited the *H. pylori* infection among the deprived population in the US (18). Second, mainstream regimens, including clarithromycin-based triple therapy consisting of a proton pump inhibitor along with clarithromycin and amoxicillin or metronidazole or bismuth-quadruple therapy (19), also effectively inhibited the transmission of the infection, when eradicating and treating *H. pylori*, regardless of gender (7). The indirect effect of successful treatment on the prevention of H. pylori was also evidenced by a cohort study during 1994–2018 (8).

On the other hand, incidence of cardia GC did not decline profoundly as non-cardia ones. The primary etiology of cardia GC is associated with gastroesophageal reflux (GER), while the secondary is relevant to *H. pylori* atrophic gastritis (6). N-nitroso compounds which are generated from the refluxate that includes a large volume of acids play a vital role in the former one. The intake of N-nitroso compounds is mainly relevant with dietary habits, lifestyle factors and GER. As a profound risk factor, GER is the most prevalent gastrointestinal disorder in the United States (20), and an increase in GER prevalence in North America and East Asia was observe in recent studies (21). These factors might together prevent a significant decline in cardia GC incidence, especially compared with non-cardia GC which are mostly arouse by *H. pylori* infection.

Interestingly, the incidence of non-cardia GC significantly increased among younger adults, while all other subgroups showed a declining tendency throughout the same period. This finding is consistent with Arnold's report (22). The decline in gastric NHL incidence was also slower among the younger adults compared with the elderly. These findings may indicate that even while infection-related gastric malignancy, non-cardia GC, and gastric NHL, occurred more often in the elderly, the transmission and morbidity of *H. pylori* in persons under 65 may continue to be cause for concern. A recent study showed that the ages of patients with *H. pylori* infection had two prevalence peaks with one at 45–49 years and the other at 70–74 years, and the detected number was higher in patients aged 45–49. More active social events, including dining together, and poor hygiene might contribute to the infection in younger people, while the higher detection rate might also be due to better implementation of early screening and diagnosis in people under 65 (23). Also, lifestyle might also play an important influence in GC onset. Younger adults are more prone to engage in excessive alcohol consumption and tobacco use, resulting in an increase or slower decline in incidence.

Despite the incidence of non-cardia GC being lower in women than in males, a significant slower decreasing trend was found. Our study also suggested that sex disparity in the incidence trend is more significant in some areas. For example, in Kentucky, the trend in non-cardia GC significantly increased for women with an AAPC of 1.5% (95% CI, 0.5–2.6%), while an opposite trend with significance was observed for men. Owning to the correlation between *H. pylori* infection and non-cardia gastric cancer, the results of this study underscored the urgency of eradicating the spread of *H. pylori* among females in some regions, despite the possibility that the disparate trends are attributable in part to the more rapid decline in smoking (another risk factor) prevalence in men than in women in the United States (24). This should raise alarm. A sex-based disproportional exposure to risk factors may differ between different regions.

The incidence in three *H. pylori*-related malignancies showed racial and ethnic disparity, with the highest incidence observed among Asians, Whites, and Hispanics for non-cardia GC, cardia GC and gastric NHL in 2019, respectively. In addition, the incidence rate of Asians in non-cardia GC declined at a significantly higher rate than other racial/ethnic groups. The possible reasons could be their communal eating practices, consumption of pickled foods, and high oil and salt intake, which might facilitate *H. pylori*'s transmission and carcinogenic process (25). However, there has been a progressive and rapid decline in the prevalence of *H. pylori* infection in Asians, which might be associated with the westernization of lifestyles (such as individual serving) and the improvements in economic conditions (26). The trend disparity in gastric NHL may be attributable to the distinctions in exposure to pathogenic factors (including *H. pylori)* by ethnicity, which was related with different employment, education, and home value (27). Furthermore, evidences showed that ethnic variation in susceptibility might existed. *H. pylori* infection led to inflammation that induces normal/abnormal immune responses, and genetic variants in the host normal/abnormal immunity was important for individual susceptibility to gastric NHL (28). Interestingly, the incidence rate of cardia GC in Whites remained at the highest and showed no significant decline in the past 20 years. This might be owing to the lack of relevance between cardia GC and *H. pylori*, and the increasing obesity epidemics, dietary habits and lifestyle factors might be the possible explanations.

Additionally, the incidence rate of non-cardia gastric cancer differed geographically. Metropolitan areas were proved to have a higher incidence rate than nonmetropolitan areas. In metropolitan regions, the incidence of gastric NHL dropped substantially more slowly. In nonmetropolitan areas, the incidence of cardia GC grew dramatically during the research period, but the incidence remained consistent in metropolitan areas. Diversities in lifestyle, diets, and underlying host genetic factors might contribute to the phenomenon. Even while improved sanitation and hygiene have reduced the intrafamilial transmission of *H. pylori* in the city to some extent, the widespread use of antibiotics on the human microbiome could contribute to the escalating problem of antibiotic resistance (29, 30). Moreover, the variety of *H. pylori*, i.e., the difference in the Cag pathogenicity island across the US, may partly contribute in part to the geographic variation. The risk of gastric cancer was proved lower among persons harboring *H. pylori* strains that lack the cag PAI. In regions with the predominance of strains harboring cagA, type s1 vacA, and other markers linked to gastric cancer, higher rate of gastric cancer might be observed (31).

Better prevention and resource allocation to reduce *H. pylori* prevalence will not only reduce the three main types of *H. pylori* infection-related cancers, but would also intervene other potential diseases caused by *H. pylori*, such as gastritis, peptic ulcer, and extra-gastroduodenal illnesses. Large-scale antibiotic use for eradication of *H. pylori* might be a cost-effective option in high-risk regions, but its adverse effects and resistance should be further clarified. Furthermore, so far there is a lack of vaccine for the prevention of *H pylori*, leaving a potential aspect to explore. Screening for pre-cancerous lesions of *H. pylori* infection-related cancers could also be profound, but no organized screening programs were massively implemented outside Asia (Japan and South Korea) (32), making the most cost-effective test-and-treat strategies for local settings still in need of illustration and testing. Future studies and efforts are warranted to accelerate *H. pylori* eradication and development new non-invasive biomarkers for *H. pylori* infection with high accuracy.

We cannot disregard the limitations in this study. First, though GC and gastric NHL is closely related to *H. pylori* infection, we cannot estimate the number of cases where the infection is pathologically confirmed because the information was not available in the SEER database. This may overestimate the incidence rate. Second, the small sample size, particularly in gastric NHL, prevents us from exploring the variations between gender and race/ethnicity in greater depth.

In summary, we have presented several significant findings. The incidence of *H. pylori*-related cancer declined dramatically in the US during 2000–2019, with the exception of cardia GC. For young adults and women, the incidence of non-cardia GC is growing and progressively declining, respectively. Also, slower decreasing trends of gastric NHL were reported for women and African Americans. The incidence rate and trend of three *H. pylori*-related cancers differed by race/ethnicity and geography, but the geographic variance for non-cardia GC was more pronounced and correlated with gender. Regarding the vital role *H. pylori* has played in the three malignancies, current international guidelines, including the American College of Gastroenterology, recommended *H. pylori* eradication in diagnosed infection, and a surveillance program, *H. pylor*i Antimicrobial Resistance Monitoring Program, was implemented nationwide (33), which might contribute to the control of *H. pylori*-related cancer. Nonetheless, to further achieve the goal of *H. pylor*i eradication, clarification of more cost-effective techniques of testing and therapy for *H. pylori* is warranted, especially the significance of screening programs and vaccines should be strengthened.

## Data availability statement

All data used in this work were publicly available, shown at: https://seer.cancer.gov.

## Ethics statement

The study was exempted from ethical review because the data were de-identified and publicly available.

## Author contributions

AW, YL, and JL designed the study. HS and ZW contributed to research data. YL, AW, YF, YB, and JL contributed to data analysis and manuscript writing. All authors contributed to supervision, manuscript revision, and gave final approval for publication.

## Funding

This work was supported by Yunnan Fundamental Research Projects (Grant Nos. 202201AU070007 and 202201AY070001-147) and Scientific Research Fund Project of Yunnan Provincial Department of Education (Grant No. 2022J0211).

## Conflict of interest

The authors declare that the research was conducted in the absence of any commercial or financial relationships that could be construed as a potential conflict of interest.

## Publisher's note

All claims expressed in this article are solely those of the authors and do not necessarily represent those of their affiliated organizations, or those of the publisher, the editors and the reviewers. Any product that may be evaluated in this article, or claim that may be made by its manufacturer, is not guaranteed or endorsed by the publisher.

## Author disclaimer

The funding agencies had no role in the design and conduct of the study; collection, management, analysis, and interpretation of the data; preparation, review, or approval of the manuscript; or decision to submit the manuscript for publication.
