# Supplementary material for: Incidence trends and disparities in Helicobacter pylori related malignancy among US adults, 2000–2019
Source: Front Public Health. 2022 Nov 28;10:1056157. doi: 10.3389/fpubh.2022.1056157 (PMC9743990; doi:10.3389/fpubh.2022.1056157)
Supplement: Supplementary file 1 [file Table_1.DOCX]

**Incidence Trends and Disparities in Helicobacter Pylori Related Malignancy among US Adults, 2000-2019**

**Supplementary Materials**

**eMethod**

**eResult**

**eTable 1**. Incidence rate and trend in *H. pylori*-related malignancies among US adults aged >= 20 years, 2000-2019, using SEER 17 database.

**eTable 2**. Incidence rate and rate and trend in non-cardia gastric cancer stratified by SEER registries.

**eTable 3**. Incidence rate and trend in cardia gastric cancer stratified by SEER registries.

**eTable 4**. Incidence rate and trend in gastric non-Hodgkin lymphoma stratified by SEER registries.

**eMethod**

GC (including cardia and non-cardia) and gastric non-Hodgkin lymphoma (NHL) cases diagnosed during 2000-2018 were included. According to the International Classification of Diseases for Oncology (ICD-O-3, third edition), GC was identified by topographic codes C16.0 to C16.6, excluding histology codes 9050-9055, 9140, 9590-9992). Cardia GC was confirmed using ICD-O-10 topographic code C16.0 and non-cardia GC were confirmed using ICD-O-10 topographic code C16.1 to C16.6. Gastric NHL was also identified by topographic code C16.0 to C16.9 and the corresponding histology code below. The incidence rate was age-adjusted, where the 2000 US population was used as the standardized population. Also, the rate was delay-adjusted to account for future corrections to the data. The adjustment strategy includes several complex methods and was detailed elsewhere (shown at: https://surveillance.cancer.gov/delay/methods.html)

| *H. Pylori* related malignancy | ICD-O-3 code | |
| --- | --- | --- |
|  | Site code | Histology code |
| Cardia gastric cancer | C16.0 | Excluding histology codes 9050-9055, 9140, 9590-9992 |
| Non-cardia gastric cancer | C16.1-C16.6 |  |
| Gastric non-Hodgkin lymphoma | C16.0-C16.9 | 9590-9597, 9670-9671, 9673, 9675, 9678-9680, 9684, 9687, 9688, 9689-9691, 9695, 9698-9702, 9705, 9708-9709, 9712, 9714-9719, 9724-9729, 9735, 9737, 9738, 9811-9818, 9823, 9827, 9837 |

**eResult**

After including GC with site code C16.8 and C16.9 in non-cardia GC, the AAPC in 2000-2019 was -1.2% (95% CI, -1.4% to -1.0%).

| **eTable 1. Incidence rate and trend in *H. pylori*-related malignancies among US adults aged >= 20 years, 2000-2019, using SEER 17 database.** | | | | | | |
| --- | --- | --- | --- | --- | --- | --- |
|  | **Case Number**  **No.** | **Incidence rate, 2000**  **(95% CI)** | **Incidence rate, 2019**  **(95% CI)** | **Average APC,**  **2000-2009**  **(95% CI)** | **Average APC,**  **2010-2019**  **(95% CI)** | **Average APC,**  **2000-2019**  **(95% CI)** |
| **Non-cardia GC** | 57,106 | 5.5 (5.3 to 5.7) | 4.3 (4.2 to 4.5) | -1.3* (-1.4 to -1.2) | -1.3* (-1.4 to -1.2) | -1.3* (-1.4 to -1.2) |
| **Cardia GC** | 33,990 | 2.9 (2.8 to 3.1) | 2.7 (2.5 to 2.8) | 0.1 (-0.3 to 0.5) | -0.8 (-1.9 to 0.3) | -0.3 (-0.9 to 0.3) |
| **Gastric NHL** | 13,213 | 1.5 (1.4 to 1.6) | 0.8 (0.7 to 0.9) | -2.9* (-3.2 to -2.6) | -2.9* (-3.2 to -2.6) | -2.9* (-3.2 to -2.6) |
| Abbreviations: GC, gastric cancer; NHL, non-Hodgkin lymphoma; APC, annual percentage change.  “*” indicates the average APC was significantly different from zero. | | | | | | |

| **eTable 2. Incidence rate and rate and trend in non-cardia gastric cancer stratified by SEER registries.** | | | | | | |
| --- | --- | --- | --- | --- | --- | --- |
| Registries | Total | | Male | | Female | |
|  | Incidence rate, 2015-2019  (95% CI) | Average APC,  2000-2019  (95% CI) | Incidence rate, 2015-2019  (95% CI) | Average APC,  2000-2019  (95% CI) | Incidence rate, 2015-2019  (95% CI) | Average APC,  2000-2019  (95% CI) |
| San Francisco-Oakland SMSA | 5.2 (4.9 to 5.6) | -1.2* (-1.8 to -0.7) | 6.2 (5.7 to 6.8) | -1.8* (-2.5 to -1.1) | 4.5 (4.1 to 4.9) | -0.5 (-1.3 to 0.3) |
| Connecticut | 4.2 (3.9 to 4.6) | -1.9* (-2.6 to -1.2) | 5.3 (4.7 to 5.8) | -2.5* (-3.5 to -1.5) | 3.5 (3.1 to 3.9) | -1.2 (-2.6 to 0.2) |
| Hawaii | 5.8 (5.2 to 6.4) | -4.5* (-5.5 to -3.6) | 7.3 (6.3 to 8.3) | -4.9* (-6.1 to -3.7) | 4.7 (4 to 5.5) | -3.9* (-5.4 to -2.5) |
| Iowa | 2.3 (2.1 to 2.6) | -1.6* (-2.7 to -0.5) | 2.7 (2.3 to 3.2) | -2.6* (-4.0 to -1.1) | 2 (1.7 to 2.4) | -0.4 (-2.0 to 1.3) |
| New Mexico | 3.7 (3.3 to 4.2) | -1.7* (-3.0 to -0.3) | 4.1 (3.5 to 4.8) | -3.0* (-4.6 to -1.4) | 3.4 (2.9 to 4) | ~ |
| Seattle (Puget Sound) | 3 (2.7 to 3.2) | -1.0* (-1.7 to -0.2) | 3.3 (2.9 to 3.7) | -2.3* (-3.3 to -1.3) | 2.8 (2.5 to 3.2) | 0.4 (-0.6 to 1.5) |
| Utah | 2.4 (2.1 to 2.7) | -2.1* (-3.5 to -0.7) | 2.9 (2.4 to 3.4) | ~ | 2 (1.6 to 2.5) | ~ |
| Atlanta (Metropolitan) | 4.8 (4.4 to 5.2) | 0.0 (-0.9 to 1.0) | 5.6 (5 to 6.3) | -0.4 (-1.4 to 0.6) | 4.2 (3.7 to 4.7) | 0.4 (-1.1 to 2.0) |
| Alaska Natives | 14.3 (10.4 to 19) | ~ | 21.1 (14 to 30.6) | ~ | 8.8 (5.1 to 14.2) | ~ |
| San Jose-Monterey | 5.4 (4.9 to 5.9) | -0.9 (-1.8 to 0.0) | 6.2 (5.5 to 7) | -1.3* (-2.5 to -0.0) | 4.8 (4.2 to 5.4) | -0.6 (-1.7 to 0.5) |
| Los Angeles | 6.7 (6.4 to 6.9) | -1.6* (-2.3 to -0.9) | 8.1 (7.6 to 8.5) | -1.8* (-2.3 to -1.3) | 5.6 (5.3 to 5.9) | -1.3* (-1.8 to -0.9) |
| Rural Georgia | 3.6 (2.3 to 5.5) | ~ | ~ | ~ | ~ | ~ |
| California excluding SF/SJM/LA | 4.3 (4.1 to 4.4) | -0.8* (-1.2 to -0.4) | 4.9 (4.7 to 5.2) | -1.5* (-1.9 to -1.0) | 3.8 (3.6 to 3.9) | 0.0 (-0.6 to 0.5) |
| Kentucky | 3.5 (3.2 to 3.7) | -0.2 (-0.9 to 0.4) | 3.8 (3.3 to 4.2) | -1.5* (-2.6 to -0.4) | 3.3 (3 to 3.7) | 1.5* (0.5 to 2.6) |
| Louisiana | 4.7 (4.4 to 5) | -1.9* (-2.7 to -1.1) | 6 (5.5 to 6.6) | -2.0* (-3.1 to -0.9) | 3.7 (3.3 to 4.1) | -2.1* (-3.1 to -1.1) |
| New Jersey | 5 (4.7 to 5.2) | -1.1* (-2.1 to -0.1) | 6 (5.7 to 6.4) | -1.6* (-2.8 to -0.5) | 4.2 (4 to 4.5) | -0.6 (-1.3 to 0.2) |
| Greater Georgia | 3.6 (3.4 to 3.9) | -0.9* (-1.6 to -0.1) | 4.1 (3.7 to 4.4) | -1.6* (-2.8 to -0.4) | 3.2 (3 to 3.6) | -0.1 (-1.0 to 0.8) |
| Idaho | 2.3 (1.9 to 2.7) | -1.1 (-2.7 to 0.4) | 2.7 (2.1 to 3.3) | ~ | 2 (1.5 to 2.5) | ~ |
| New York | 5.9 (5.8 to 6.1) | 0.0 (-0.4 to 0.4) | 7 (6.7 to 7.3) | -0.5* (-1.0 to 0.0) | 5.1 (4.9 to 5.4) | 0.4 (-0.2 to 0.9) |
| Massachusetts | 3.7 (3.5 to 4) | -2.1* (-2.6 to -1.5) | 4.7 (4.3 to 5.1) | -2.9* (-3.9 to -1.9) | 3 (2.7 to 3.3) | -1.2* (-2.0 to -0.4) |
| Illinois | 4.2 (4 to 4.3) | -0.5 (-1.0 to 0.0) | 5 (4.7 to 5.3) | -1.1* (-1.6 to -0.5) | 3.5 (3.3 to 3.8) | 0.1 (-0.6 to 0.8) |
| Texas | 3.9 (3.8 to 4) | -0.3 (-0.7 to 0.1) | 4.7 (4.5 to 4.9) | -1.0* (-1.5 to -0.6) | 3.3 (3.1 to 3.5) | 0.4 (-0.2 to 0.9) |
| APC, annual percentage change.  “^” indicates the observed number of non-cardia gastric cancer diagnosis was fewer than 16 and the incidence was not estimated.  “~” indicates the observed number of non-cardia gastric cancer diagnosis was fewer than 16 in any year of the period and the incidence of that was not estimated. Thus, Joinpoint regression and APC analysis could not be performed.  “*” indicates the average APC was significantly different from zero. | | | | | | |

| **eTable 3. Incidence rate and trend in cardia gastric cancer stratified by SEER registries.** | | | | | | |
| --- | --- | --- | --- | --- | --- | --- |
| Registries | Total | | Male | | Female | |
|  | Incidence rate, 2015-2019  (95% CI) | Average APC,  2000-2019  (95% CI) | Incidence rate, 2015-2019  (95% CI) | Average APC,  2000-2019  (95% CI) | Incidence rate, 2015-2019  (95% CI) | Average APC,  2000-2019  (95% CI) |
| San Francisco-Oakland SMSA | 2.6 (2.4 to 2.9) | -0.7 (-1.7 to 0.3) | 4.4 (3.9 to 4.8) | -1.2* (-2.2 to -0.2) | 1.2 (1.0 to 1.5) | ~ |
| Connecticut | 3.8 (3.5 to 4.1) | -0.5 (-2.2 to 1.1) | 6.5 (5.9 to 7.1) | -1.3 (-3.3 to 0.7) | 1.6 (1.3 to 1.9) | 1.5 (-0.1 to 3.0) |
| Hawaii | 2.4 (2.1 to 2.9) | -1.0 (-2.8 to 0.8) | 4.1 (3.4 to 4.9) | ~ | 0.9 (0.6 to 1.3) | ~ |
| Iowa | 2.9 (2.6 to 3.2) | -0.5 (-1.4 to 0.5) | 4.7 (4.2 to 5.2) | -1.0 (-2.1 to 0.0) | 1.4 (1.1 to 1.7) | ~ |
| New Mexico | 1.9 (1.6 to 2.2) | -0.7 (-2.6 to 1.2) | 3.0 (2.5 to 3.6) | -1.5 (-3.5 to 0.5) | 0.9 (0.7 to 1.2) | ~ |
| Seattle (Puget Sound) | 3.5 (3.2 to 3.7) | -0.2 (-0.9 to 0.5) | 5.6 (5.1 to 6.1) | -0.6 (-1.5 to 0.3) | 1.6 (1.4 to 1.9) | 0.9 (-0.4 to 2.2) |
| Utah | 2.4 (2.1 to 2.8) | 0.7 (-0.8 to 2.2) | 4.1 (3.5 to 4.8) | ~ | 0.9 (0.7 to 1.2) | ~ |
| Atlanta (Metropolitan) | 2.3 (2 to 2.5) | 1.1 (-0.4 to 2.6) | 4.1 (3.5 to 4.7) | ~ | 0.8 (0.6 to 1.1) | ~ |
| Alaska Natives | ~ | ~ | ~ | ~ | ~ | ~ |
| San Jose-Monterey | 2.7 (2.3 to 3) | -0.4 (-1.2 to 0.3) | 4.4 (3.8 to 5.0) | -0.8 (-1.8 to 0.2) | 1.1 (0.9 to 1.5) | ~ |
| Los Angeles | 2.3 (2.2 to 2.5) | -1.2* (-2.1 to -0.3) | 3.9 (3.6 to 4.2) | -1.4* (-2.3 to -0.4) | 1.0 (0.9 to 1.2) | -1.2 (-2.0 to 0.0) |
| Rural Georgia | ~ | ~ | ~ | ~ | ~ | ~ |
| California excluding SF/SJM/LA | 2.7 (2.6 to 2.8) | -0.5* (-0.9 to -0.1) | 4.5 (4.3 to 4.7) | -0.7* (-1.2 to -0.2) | 1.1 (1.0 to 1.2) | -0.1(-0.9 to 0.7) |
| Kentucky | 3.5 (3.2 to 3.8) | 2.4* (1.3 to 3.4) | 5.9 (5.4 to 6.5) | 2.0* (0.6 to 3.4) | 1.4 (1.2 to 1.6) | ~ |
| Louisiana | 2.7 (2.5 to 3.0) | 0.4 (-0.4 to 1.2) | 4.6 (4.1 to 5.1) | 0.1 (-0.8 to 1.0) | 1.2 (1.0 to 1.4) | ~ |
| New Jersey | 3.1 (3.0 to 3.3) | -0.4 (-0.9 to 0.1) | 5.3 (4.9 to 5.6) | -0.5 (-1.1 to 0.2) | 1.4 (1.2 to 1.6) | -0.5 (-1.4 to 0.3) |
| Greater Georgia | 2.4 (2.2 to 2.6) | 1.0* (0.2 to 1.8) | 4.1 (3.8 to 4.5) | 0.4 (-0.4 to 1.2) | 1.0 (0.8 to 1.2) | ~ |
| Idaho | 3.1 (2.7 to 3.5) | 0.4 (-1.2 to 2.1) | 5.3 (4.5 to 6.2) | ~ | 1.1 (0.8 to 1.5) | ~ |
| New York | 3.4 (3.2 to 3.5) | -0.2 (-0.7 to 0.3) | 5.6 (5.4 to 5.9) | -0.3 (-0.8 to 0.2) | 1.5 (1.4 to 1.6) | -0.4 (-1.2 to 0.5) |
| Massachusetts | 3.1 (2.9 to 3.3) | -0.1 (-1.8 to 1.6) | 5.1 (4.7 to 5.5) | 0.2 (-0.6 to 0.9) | 1.5 (1.3 to 1.7) | 2.1* (0.8 to 3.4) |
| Illinois | 3.2 (3.1 to 3.4) | 0.6 (-0.8 to 2.2) | 5.5 (5.2 to 5.8) | 0.9 (-1.3 to 3.2) | 1.4 (1.2 to 1.5) | 1.7* (0.7 to 2.7) |
| Texas | 2.2 (2.1 to 2.3) | -0.5 (-1.1 to 0.2) | 3.7 (3.5 to 3.9) | -0.7 (-1.4 to 0.1) | 0.9 (0.8 to 1.0) | -0.4 (-1.2 to 0.4) |
| APC, annual percentage change.  “^” indicates the observed number of non-cardia gastric cancer diagnosis was fewer than 16 and the incidence was not estimated.  “~” indicates the observed number of non-cardia gastric cancer diagnosis was fewer than 16 in any year of the period and the incidence of that was not estimated. Thus, Joinpoint regression and APC analysis could not be performed.  “*” indicates the average APC was significantly different from zero. | | | | | | |

| **eTable 4. Incidence rate and trend in gastric non-Hodgkin lymphoma stratified by SEER registries.** | | | | | | |
| --- | --- | --- | --- | --- | --- | --- |
| Registries | Total | | Male | | Female | |
|  | Incidence rate, 2015-2019  (95% CI) | Average APC,  2000-2019  (95% CI) | Incidence rate, 2015-2019  (95% CI) | Average APC,  2000-2019  (95% CI) | Incidence rate, 2015-2019  (95% CI) | Average APC,  2000-2019  (95% CI) |
| San Francisco-Oakland SMSA | 0.9 (0.8 to 1.0) | -2.5* (-3.7 to -1.4) | 1.1 (0.9 to 1.4) | ~ | 0.7 (0.6 to 0.9) | ~ |
| Connecticut | 1.2 (1.0 to 1.4) | -1.1 (-2.9 to 0.7) | 1.3 (1.0 to 1.6) | ~ | 1.1 (0.9 to 1.4) | ~ |
| Hawaii | 1.1 (0.9 to 1.4) | ~ | 1.6 (1.2 to 2.2) | ~ | 0.7 (0.4 to 1.0) | ~ |
| Iowa | 0.6 (0.5 to 0.8) | ~ | 0.7 (0.5 to 1.0) | ~ | 0.5 (0.4 to 0.7) | ~ |
| New Mexico | 0.7 (0.5 to 0.9) | ~ | 0.8 (0.6 to 1.2) | ~ | 0.6 (0.4 to 0.8) | ~ |
| Seattle (Puget Sound) | 0.9 (0.8 to 1.1) | -2.8* (-4.3 to -1.3) | 1.2 (1.0 to 1.5) | ~ | 0.7 (0.5 to 0.9) | ~ |
| Utah | 0.5 (0.4 to 0.6) | ~ | 0.7 (0.5 to 1.0) | ~ | ~ | ~ |
| Atlanta (Metropolitan) | 0.8 (0.6 to 0.9) | ~ | 0.9 (0.6 to 1.2) | ~ | 0.7 (0.5 to 0.9) | ~ |
| Alaska Natives | ~ | ~ | ~ | ~ | ~ | ~ |
| San Jose-Monterey | 0.9 (0.7 to 1.1) | ~ | 1 (0.7 to 1.3) | ~ | 0.8 (0.6 to 1.1) | ~ |
| Los Angeles | 1.0 (0.9 to 1.1) | -3.5* (-4.2 to -2.2) | 1.1 (1.0 to 1.3) | -3.6* (-5.1 to -2.1) | 0.9 (0.8 to 1.1) | -2.8* (-4.2 to -1.3) |
| Rural Georgia | ~ | ~ | ~ | ~ | ~ | ~ |
| California excluding SF/SJM/LA | 0.9 (0.8 to 0.9) | -2.6* (-3.1 to -2.1) | 1.1 (1.0 to 1.2) | -2.4* (-3.0 to -1.8) | 0.7 (0.6 to 0.8) | -2.9* (-3.7 to -2.1) |
| Kentucky | 0.8 (0.7 to 0.9) | -5.2* (-7.5 to -2.7) | 1.0 (0.8 to 1.3) | ~ | 0.6 (0.5 to 0.8) | ~ |
| Louisiana | 1.0 (0.8 to 1.1) | -3.2* (-5.3 to -1.1) | 1.2 (1.0 to 1.5) | -4.7* (-7.4 to -2.0) | 0.8 (0.6 to 1.0) | ~ |
| New Jersey | 1.1 (1.0 to 1.3) | -2.1* (-3.0 to -1.2) | 1.5 (1.3 to 1.7) | -2.3* (-3.7 to -0.9) | 0.9 (0.8 to 1.1) | -1.9* (-2.9 to -1.0) |
| Greater Georgia | 0.8 (0.7 to 0.9) | -3.3* (-4.5 to -2.1) | 1.0 (0.9 to 1.2) | -4.0* (-5.3 to -2.7) | 0.7 (0.5 to 0.8) | ~ |
| Idaho | 0.7 (0.5 to 1.0) | ~ | 1.0 (0.7 to 1.5) | ~ | ~ | ~ |
| New York | 1.1 (1.1 to 1.2) | -1.5* (-2.1 to -0.9) | 1.4 (1.3 to 1.5) | -1.6* (-2.5 to -0.6) | 1.0 (0.9 to 1.1) | -1.5* (-2.3 to -0.7) |
| Massachusetts | 0.9 (0.8 to 1.0) | -2.0* (-3.1 to -0.9) | 1.0 (0.9 to 1.2) | -2.5* (-4.2 to -0.9) | 0.7 (0.6 to 0.9) |  |
| Illinois | 1.0 (0.9 to 1.1) | -1.4* (-2.2 to -0.5) | 1.3 (1.1 to 1.4) | -1.6* (-2.5 to -0.6) | 0.8 (0.7 to 0.9) | -1.3* (-2.4 to -0.1) |
| Texas | 0.8 (0.8 to 0.9) | -2.9* (-4.5 to -1.3) | 1.0 (1.0 to 1.2) | -3.2* (-4.4 to -2.0) | 0.7 (0.6 to 0.7) | -3.3 (-8.1 to 1.7) |
| APC, annual percentage change.  “^” indicates the observed number of non-cardia gastric cancer diagnosis was fewer than 16 and the incidence was not estimated.  “~” indicates the observed number of non-cardia gastric cancer diagnosis was fewer than 16 in any year of the period and the incidence of that was not estimated. Thus, Joinpoint regression and APC analysis could not be performed.  “*” indicates the average APC was significantly different from zero. | | | | | | |
